# Daily Time-Use Patterns and Obesity and Mental Health among Primary School Students in Shanghai: A Population-Based Cross-Sectional Study

**DOI:** 10.1038/s41598-017-15102-4

**Published:** 2017-11-23

**Authors:** Yunting Zhang, Donglan Zhang, Xinyue Li, Patrick Ip, Frederick Ho, Yanrui Jiang, Wanqi Sun, Qi Zhu, Weiming Zhu, Jun Zhang, Hongyu Zhao, Guanghai Wang, Xiaoming Shen, Fan Jiang

**Affiliations:** 1 Child Health Advocacy Institute, Shanghai Children’s Medical Center, Shanghai Jiao Tong University School of Medicine, Shanghai, China; 2 School of Public Health, Shanghai Jiao Tong University, Shanghai, China; 3 Department of Health Policy and Management, College of Public Health, University of Georgia, Athens, GA USA; 4 Department of Biostatistics, School of Public Health, Yale University, New Heavens, CT USA; 5 Department of Paediatrics and Adolescent Medicine, University of Hong Kong, Queen Mary Hospital, 102 Pok Fu Lam Road, Hong Kong, China; 6 Department of Developmental and Behavioral Pediatrics, Shanghai Children’s Medical Center, Shanghai Jiao Tong University School of Medicine, Shanghai, China; 7 China Center for Health Development Studies, Peking University, Beijing, China; 8 Ministry of Education-Shanghai Key Laboratory of Children’s Environmental Health, Shanghai, China; 9 Shanghai Jiao Tong University – Yale Joint Center for Biostatistics, Shanghai, China

## Abstract

Physical activity, screen viewing, sleep, and homework among children have been independently linked to health outcomes. However, few studies have assessed the independent associations between time spent in daily activities and children’s physical and mental health. This study describes time spent in four activities among primary school students in Shanghai, and examines the relationship between daily time-use patterns and obesity and mental health. The representative sample consists of 17,318 children aged 6–11 years in Shanghai. Time spent in moderate to vigorous physical activities (MVPA), screen viewing, sleep, and homework was measured by validated questionnaires. Logistic regressions were performed. We also fitted generalized additive models (GAM) and performed two-objective optimization to minimize the probability of poor mental health and obesity. In 2014, 33.7% of children spent ˂1 hour/day on MVPA, 15.6% spent ≥ 2 hours/day on screen viewing, 12.4% spent ˂ 9 hours/day on sleep, and 27.2% spent ≥ 2 hours/day on homework. The optimization results suggest that considering the 24-hour time limit, children face trade-offs when allocating time. A priority should be given to the duration of sleep and MVPA. Screen exposure should be minimized to save more time for sleep and other beneficial activities.

## Introduction

A burgeoning literature has linked moderate to vigorous physical activity (MVPA) to improved health outcomes in children and adolescents^1–3^. MVPA generates more energy expenditure, reduces children’s cardio-metabolic risk factors, and improves mood, attention, cognition and academic performance^3–8^. In contrast, sedentary behaviors such as television viewing, video-game playing, computer use, and in some cases, excessive time spent on homework have been shown to be related to adverse health outcomes^9–11^, potentially offsetting the beneficial effects of MVPA on health^12^. One can be both active and sedentary. Vice-versa, one can also be inactive but not sedentary. These two dimensions have been independently related to health outcomes^13^. In addition to inadequate MVPA and sedentary lifestyles, evidence also supports a relationship between short sleep duration and increased risk of childhood obesity^14,15^, as well as behavioral and depressive symptoms^16^. Although evidence linking behaviors and health is strong, most studies to date have focused on the relationship between time spent in a single activity and health outcomes. Guidelines about children’s time use have been proposed separately for each behavior without the benefit of analyses controlling for time spent in multiple activities. Specifically, the World Health Organization recommends at least 60 minutes of MVPA daily for children aged 5–17 years^17^, the American Academy of Pediatrics (AAP) recommends less than 2 hours of screen viewing for children and adolescents^18^, the United States (US) National Sleep Foundation recommends a 9–11 hour sleep duration for school-aged children^19^, and the US National Education Association recommends 10 to 50 minutes of homework per day for students in grades 1 through 5^20^. Yet, time use is determined across several categories such that greater time use in one activity may mean less time use in another activity. It is important, therefore, to obtain estimates of the associations between time use in multiple activities and health outcomes and to further examine the combined associations. Previous studies have examined the combination of behaviors associated with children’s health^21,22^. However, those studies were limited because they defined daily activities based on energy expenditure rather than time allocation to specific behaviors.

For school children, time allocation during school is relatively fixed by the curriculum. Children’s major activities after school include MVPA, screen viewing, homework and sleep, all of which have been related to health outcomes. While Shanghai’s education model has drawn global attention for its top ranking in the Program for International Student Assessment (PISA)^23^, concerns about declining physical fitness and overall health in children have increased. The obesity prevalence of Shanghai’s primary school students was almost as high as that of children in developed countries^24^. Studies have also reported that Chinese students experienced high levels of academic stress^25^. It is therefore warranted to evaluate lifestyles, behaviors, and health status in these children. Using a population-based survey conducted among primary school students in Shanghai, this study aims to (1) assess the distribution of children’s daily time spent in MVPA, screen viewing, homework, and sleep; (2) evaluate the combined associations between daily time use in several activities and obesity and poor mental health, two most pressing public health issues affecting child development in Chinese children.

## Methods

### Study design and data collection

We conducted the Shanghai Children’s Health, Education and Lifestyle Evaluation (SCHEDULE) study in July, 2014. The SCHEDULE was designed to represent children aged 6 to 11 years (grades 1 to 5) in Shanghai’s primary schools. We excluded students with special needs or at international schools. The SCHEDULE investigated a broad range of questions including socio-demographic characteristics, health behaviors (i.e., physical activity, sleep duration, and sedentary behaviors), health status (i.e., body mass index (BMI), self-reported health problems, mental health status), and academic performances.

We selected study participants using a multistage cluster sampling approach. There are 16 districts and 1 county in Shanghai. Six districts and 1 county were randomly selected to represent the entire city. Second, we randomly selected 26 public primary schools, the primary sampling units (PSUs), from the 7 districts/county in proportion to population size. Third, we stratified the sampled schools by school size. All students from grades 1 to 5 were selected if a school had less than 1000 students, and half of the students in a school were selected if the number of students was above 1000, with random selection of class numbers. Finally, we conducted interviews among the sampled students as well as one of their parents and the head teacher of their classes. The initial sample consisted of 17,624 participants, and 17,318 of them answered the questionnaires. Sampling weights were computed using inverse probability weighting, which represented the inverse of the combined selection probability for each stage, including the non-response rate (1.74%). Details of the sampling procedures and quality control strategies were presented in Appendix 1.

The study was approved by the institutional review board of the Shanghai Children’s Medical Center (SCMC), Shanghai Jiao Tong University (SCMCIRB-K2014033). All methods were performed in accordance with the relevant guidelines and regulations. All parents and teachers of the children who participated in the study gave written informed consent.

### Measurements

Time spent in MVPA, screen viewing, and homework during weekdays and weekends was measured using the Chinese version of the International Children’s Leisure Activities Study Survey Questionnaire (CLASS-C) which was validated in Hongkong population^26^. CLASS-C is a 7-day recall questionnaire used to measure MVPA and sedentary behaviors among school-aged children and adolescents^26^. Studies found that the CLASS-C had good test-retest reliability and acceptable criterion validity when compared with accelerometer-measured MVPA^26^. Screen viewing included watching television, playing video games, and using computers. Homework included assignments from both schools and extracurricular studies. We first derived the average time spent in MVPA, screen viewing, and homework per day, then categorized the variables as ˂1 hour, 1–2 hours, and ≥2 based on the current guidelines and their distributions in the population.

For sleep duration, children’s bedtimes and waking times on weekdays and weekends during the past month were collected from parental report (e.g., ‘*what was the usual bedtime on weekdays during the past month? What was the usual bedtime on weekends during the past month?’*). The average sleep duration was calculated using the formula: ([weekday sleep duration × 5] + [weekend sleep duration × 2])/7. This sleep questionnaire produces estimates reasonably consistent with objectively-measured sleep duration by actigraphy (r = 0.59–0.74)^27^.

Anthropometric data including height and weight were measured three times by the same group of medical staff from the SCMC. The average measures were used to compute the BMI by dividing the weight in kilograms by the square of the height in meters. Obesity status was determined based on sex specific BMI-for-age cutoff standard for Chinese children^28^.

Children’s mental health status was ascertained with 25 questions in the Chinese version of the parent-reported Strengths and Difficulties Questionnaire (SDQ), a brief behavioral and psychosocial wellbeing screening questionnaire for children aged 3 to 16 years^29,30^. Previous studies confirmed the questionnaire’s reliability and validity among primary school children in China^31,32^. Poor mental health was defined based on the cutoff scores of the total difficulties (SDQ ≥ 17)^33^.

Controlled variables associated with obesity and poor mental health included children’s age (<8, 8–10, ≥10 years), gender and health status (parent-reported having any chronic health problems such as asthma, rhinitis and etc.), household income (<30,000 Renmingbi (RMB), 30,000–100,000 RMB, ≥100,000 RMB) and parents’ highest educational attainment (middle school and below, high school, college and above).

For the model using obesity as an outcome, paternal and maternal BMI and children’s dietary patterns were further controlled. Children’s dietary patterns were evaluated with a modified food frequency questionnaire (FFQ) with nine food items^34^. Parents were asked to estimate the frequency with which their children consumed these foods during the past month by selecting one of three responses: 0–2 times a week, 3–5 times a week, or 6 or more times a week. Factor analysis was used to derive two main factors describing the dietary patterns: “healthy dietary factor score” and “unhealthy dietary factor score.”^35^ Details about the FFQ analysis were presented in Appendix 2.

### Data analysis

Descriptive analyses were initially conducted to assess the percentage of missing values in all variables. We performed multiple imputations five times with a chained approach and separate regression model for each variable (missing value patterns and imputation process were presented in Appendix 1). Using the multiply imputed sample, we first described the average time spent in MVPA, screen viewing, homework, and sleep per day, the distributions of these four time variables, and the categorical demographic variables by the two health outcomes. We applied sampling weights in all analyses. Next, survey-weighted logistic regressions were used to estimate the simultaneous associations between the time spent in each activity and obesity and poor mental health after adjusting for age, gender, annual household income, parents’ highest educational attainment, and the presence of health problems. In the regression with obesity as the outcome, dietary patterns and parents’ BMI were further added as covariates. Finally, we fitted generalized additive models (GAM) using the same covariates as in the logistic regression models and performed two-objective optimization to minimize the probability of poor mental health and obesity. We chose GAM because it extends logistic regression to allow for non-linear association and serves as useful tools for finding the optimal time allocation strategies. In the optimization procedure, we gave equal weights to the two probabilities and minimize the sum of the two as we viewed the two outcomes equally important. We also considered the trade-off between activities that the increased time in one activity necessarily decreases the time spent in other activities, and therefore we set the sum of the four time variables (sleep, MVPA, screen, and homework) as fixed and searched for the optimal time allocation strategy. Further, because the total time of the four variables ranges between 10 to 15 hours in 90% of our data, we performed optimization under the sum constraint at 10, 11,…, 15  hours respectively and provided the optimal time allocation strategy under each time constraint. Details about the optimization analysis were presented in Appendix 3. Descriptive and regression analyses were performed using STATA 14.2 (StataCorp LP, College Station, TX). Odds ratios (ORs) with 95% confidence intervals (CIs) were reported. Sampling weights were used in all analyses to ensure representativeness. Two-objective optimization was performed in R (version 3.3.2) and R package mgcv was used^36^.

### Data Availability

The datasets generated during and/or analyzed during the current study are available from the corresponding author on reasonable request.

## Results

The prevalence of obesity in Shanghai’s primary school students was 10.3% (Table 1). For children who were obese, the distribution of MVPA time did not vary significantly by weight status. However, ≥2 hours of screen time was more prevalent among those who were obese (19.8% vs. 15.1%, p = 0.001). Also, the percentage of children who had sleep duration of < 9 hour was significantly higher in those who were obese (16.5% vs. 12.0%, p < 0.001) and so was the percentage of children spent ≥ 2 hours on homework (31.2% vs. 26.8%, p < 0.05). Boys, children with health problems, children whose parents had higher BMI and children with higher unhealthy dietary scores were more likely to be obese (p < 0.05).

**Table 1:** Sample Characteristics by Body Weight and Mental Health Status, 2014 Shanghai Children’s Health, Education and Lifestyle Evaluation (n = 17318)^a^.

	Total	Weight Status^b^			Mental Health Status^e^		
		Not obese	Obese	*P*-value^e^	Good	Poor	*P*-value^e^
		89.70%	10.30%		88.70%	11.30%	
Moderate to vigorous physical activity (hours/day)							
Mean (SE)	1.69 (0.01)	1.68 (0.01)	1.71 (0.05)	0.632	1.72 (0.02)	1.42 (0.04)	<0.001
<1	33.70%	33.60%	34.60%	0.307	32.30%	44.70%	<0.001
1–2	37.40%	37.60%	35.70%		37.80%	34.30%	
≥2	28.90%	28.80%	29.70%		29.90%	21.00%	
Screen viewing (hours/day)							
Mean (SE)	1.10 (0.02)	1.07 (0.02)	1.33 (0.06)	<0.001	1.08 (0.02)	1.23 (0.06)	0.025
<1	60.70%	61.40%	54.10%	<0.001	61.10%	57.50%	0.017
1–2	23.70%	23.40%	26.10%		23.60%	24.30%	
≥2	15.60%	15.10%	19.80%		15.30%	18.20%	
Sleep duration (hours/day)							
Mean (SE)	9.60 (0.01)	9.61 (0.01)	9.51(0.02)	<0.001	9.62 (0.01)	9.50 (0.02)	<0.001
<9	12.40%	12.00%	16.50%	<0.001	11.70%	18.40%	0.001
9–10	63.20%	63.00%	65.00%		63.60%	60.40%	
≥10	24.30%	25.00%	18.50%		24.70%	21.20%	
Homework (hours/day)							
Mean (SE)	1.27 (0.01)	1.26 (0.02)	1.37 (0.05)	0.018	1.28 (0.02)	1.22 (0.04)	0.391
<1	50.70%	51.10%	47.10%	0.003	50.10%	55.50%	0.195
1–2	22.10%	22.10%	21.70%		22.80%	16.20%	
≥2	27.20%	26.80%	31.20%		27.10%	28.40%	
Age				0.287			<0.001
Mean (SE)	9.18	9.19	9.13		9.21	9	
	-0.02	-0.02	-0.05		-0.02	-0.05	
Gender				<0.001			<0.001
Boys	53.40%	51.80%	66.80%		51.90%	64.60%	
Girls	46.60%	48.20%	33.20%		48.10%	35.40%	
Annual family income ^d^				0.957			<0.001
Low income	10.20%	10.20%	9.90%		9.60%	14.80%	
Middle income	43.60%	43.50%	44.00%		42.70%	50.40%	
High income	46.20%	46.30%	46.10%		47.70%	34.80%	
Parent’s highest education level							
Middle school and below	21.70%	21.90%	19.60%	0.11	20.60%	29.60%	<0.001
High school	29.20%	29.20%	29.50%		28.80%	32.60%	
College and above	49.10%	48.90%	50.90%		50.60%	37.90%	
Having health problems				0.007			0.001
No	84.20%	84.60%	81.30%		84.70%	80.50%	
Yes	15.80%	15.40%	18.70%		15.30%	19.50%	
Mother’s body mass index				<0.001			
	21.67 (0.03)	21.56 (0.04)	22.62 (0.10)		/	/	
Father’s body mass index				<0.001			
	24.11 (0.04)	23.99 (0.04)	25.18 (0.12)		/	/	
Healthy dietary factor score				0.779			
	0.01 (0.01)	0.01 (0.01)	0.00 (0.03)		/	/	
Unhealthy dietary factor score				0.003			
	−0.03 (0.01)	−0.04 (0.01)	0.06 (0.03)		/	/	

a After multiple imputations, sampling weights were used in all analyses. Results were presented as means (standard errors)/percentages.

b Weight status was defined based on the BMI-for-age percentile cutoff standards in Chinese children.

c Mental health was measured by the Chinese version of the Strengths and Difficulties Questionnaire (SDQ). Poor mental health was defined as SDQ score ≥17.

d Annual family income was categorized into four groups: low-income (<30,000 RMB, reference group), middle-low income (30,000–100,000 RMB), middle-income (100,000–300,000 RMB), and high-income (300,000 RMB and above).

e *P*-values were computed using Chi-square tests.

Table 2 presents the association between time-use categories and obesity, after adjusting for related covariates. The odds ratio of time spent in each activity compared with the current guideline-recommended time (MVPA: 1–2 hours; screen time:<1 hour; sleep duration:≥10 hours; homework:<1 hour) shows that among time spent in the four lifestyle behaviors, insufficient sleep had the largest magnitudes of association with both obesity and poor mental health. In the obesity model, screen viewing for over 2 hours per day was associated with higher odds of obesity (OR = 1.38, 95% CI: 1.14, 1.66). Less than 9 hours of sleep predicted higher odds of being obese (OR = 1.91, 95% CI: 1.50, 2.43). However, time spent in MVPA and homework was not associated with the odds of obesity.

**Table 2:** Associations Between Children’s Daily Time Use and Weight and Mental Health Status, 2014 Shanghai Children’s Health, Education and Lifestyle Evaluation (n = 17318) ^a^.

		Obesity^b^	Poor Mental Health^c^
		OR (95% CI)	OR (95% CI)
Moderate to vigorous physical activity (Reference: 1,2 hours/day)			
<1 hour/day		1.06 (0.90, 1.26)	1.37** (1.15, 1.64)
≥2 hours/day		1.08 (0.91, 1.29)	0.78* (0.63, 0.95)
Screen viewing (Reference:<1 hour/day)			
1–2 hours/day		1.21* (1.03, 1.43)	1.09 (0.93, 1.29)
≥2 hours/day		1.38** (1.14, 1.66)	1.28* (1.05, 1.55)
Sleep duration (Reference:≥10 hours/day)			
<9 hour/day	1.91*** (1.50, 2.43)		2.17*** (1.71, 2.76)
9–10 hours/day	1.43*** (1.23, 1.67)		1.18* (1.00, 1.39)
Homework (Reference:<1 hour/day)			
1–2 hours/day	1.01 (0.78, 1.31)		0.70*** (0.57, 0.85)
≥2 hours/day	1.11 (0.94, 1.31)		1.02 (0.86, 1.20)

aAfter multiple imputations, sampling weights were used in all analyses. Results were presented as odds ratios (95% confidence intervals). Models estimated the association between time spent in each activity and obesity and mental health outcomes, adjusting for age groups, sex, annual household income, parent’ highest education level, and health problems. In the obesity model, we further adjusted for father and mother’s body mass index and children’s diet-related factor scores.

b Obesity was defined based on the BMI-for-age percentile cutoff standards in Chinese children.

c Mental health was measured by the Chinese version of the Strengths and Difficulties Questionnaire (SDQ). Poor mental health was defined as SDQ score ≥17.

*P < 0.05, **P < 0.01, ***P < 0.001.

The prevalence of poor mental health in Shanghai’s primary school students was 11.3% (Table 1). Among children who had poor mental health, we observed that a higher percentage of children spent ˂ 1 hour on MVPA (44.7% vs. 32.3%, p < 0.001). Also, compared to children with good mental health, more children with poor mental health had ≥ 2 hours of screen time (18.2% vs. 15.3%, p = 0.011) and had sleep duration of ˂9 hours (18.4% vs 11.7%, p < 0.001). Boys, children at younger age, children with health problems, children whose parents had a lower educational level and those from families with lower household income were more likely to have poor mental health (p < 0.001).

In the logistic regression model for mental health, compared with 1–2 hours of MVPA, less than 1 hour of MVPA was associated with significantly higher odds of poor mental health (OR = 1.37, 95% CI: 1.15, 1.64). Same was found for over 2 hours of screen viewing (OR = 1.28, 95% CI: 1.05, 1.55) and less than 9 hours of sleep duration (OR = 2.17, 95% CI: 1.71, 2.76). Doing homework for 1–2 hours per day was significantly associated with lower odds of poor mental health compared with ˂ 1 hour of homework (OR = 0.70, 95% CI: 0.57, 0.85) (Table 2).

**Figure 1:**
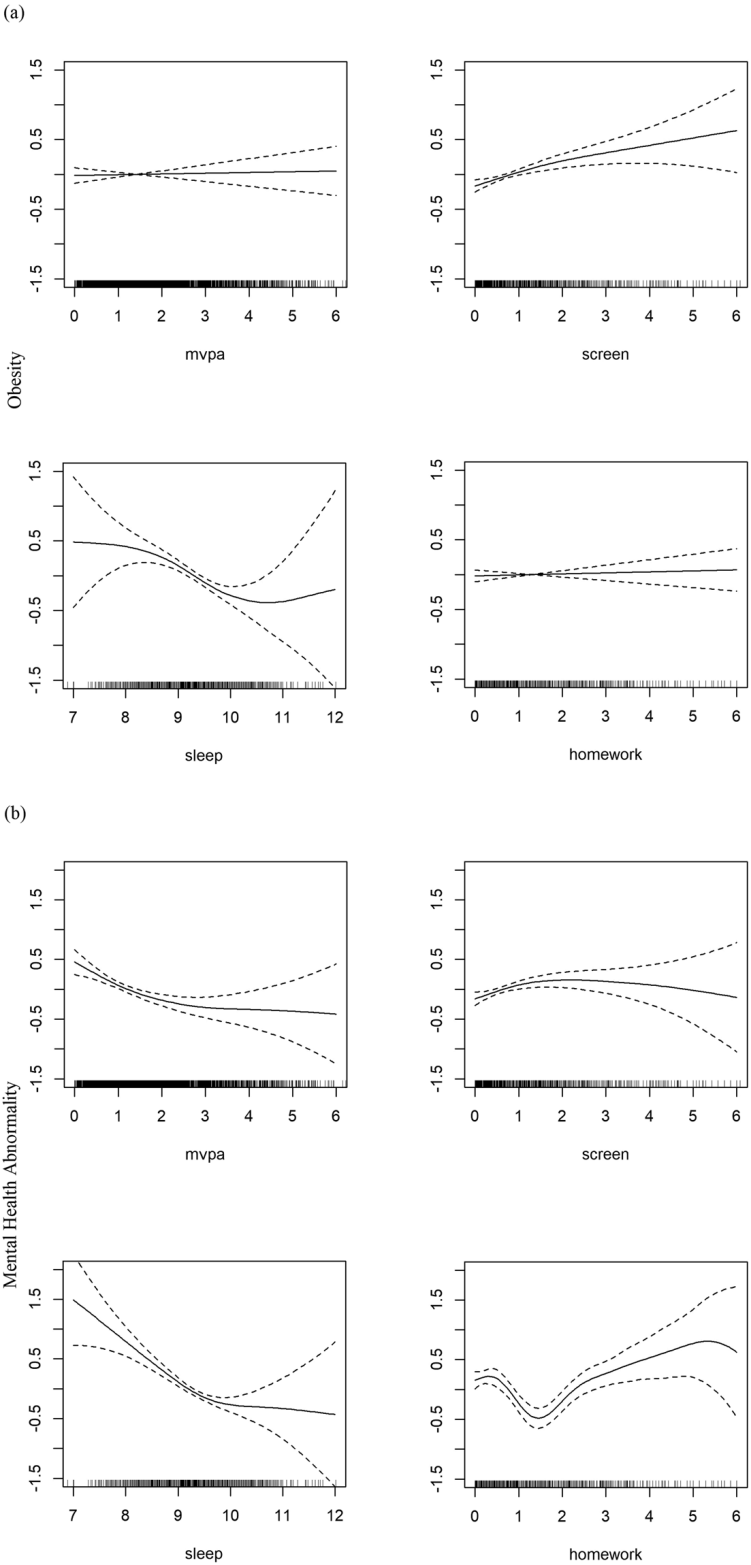
The GAM fitted smooth functions for the association between the four time variables and the two outcomes respectively. Solid line - the estimated smooth function on the scale of the linear predictor. Dotted line - the 95% confidence interval for the estimated function. Bottom - rug plot showing the distribution of the covariate.

Figure 1(a) shows the GAM fitted smooth functions for the association between the four time variables and obesity. As the GAM analysis suggests, obesity was strongly associated with screen exposure and duration of sleep but not with MVPA and homework. An increase in the time of screen exposure led to an increase in the probability of obesity. As the duration of sleep increased from 7 to around 11 hours, the probability of obesity decreased.

Figure 1(b) shows the GAM fitted smooth functions for the association between the four time variables and mental health, and poor mental health was significantly associated with all four variables. MVPA and sleep were both negatively associated with poor mental health, but the strength of association varied over different values of the time variables. Increasing the time spent in MVPA from 0 to 2 hours and increasing the duration of sleep from 8 to 10 hours led to a decrease in the probability of having mental health problems. However, a further increase in time spent in either activity would not be as effective in decreasing the probability, as the estimated function suggested a smaller slope and a weaker effect. For screen exposure, its association with poor mental health was moderately significant (p-value 0.025). If the time of screen exposure increased from 0 to 2 hours, the probability of having mental health problems increased. Spending too little time and spending too much time on homework were both not beneficial for mental health. Allocating 1 to 2 hours to homework led to a relatively low probability of poor mental health.

Therefore, in the two-objective optimization based on GAM, as the total time increased, the extra time was first allocated to the duration of sleep to ensure enough sleep (Table 3). The extra time was further added to MVPA and homework. After enough sleep was guaranteed, allocating the extra time to MVPA was preferred. Screen exposure should be minimized at all times to save more time for sleep and other beneficial activities.

**Table 3:** Optimal Time Allocation Strategies from the Two-Objective Optimization for Different Time Constraints (10 to 15 hours).

Time Constraint (hours)	Sleep Duration (hours)	Moderate to Vigorous Physical Activity Time (hours)	Screen Time (hours)	Homework Time (hours)
10	8	0.5	0.5	1
11	9	0.5	0.5	1
12	10	0.5	0.5	1
13	10	1.0	0.5	1.5
14	10.5	1.5	0.5	1.5
15	10.5	2.5	0.5	1.5

## Discussion

Using a representative sample, our study found primary school students in Shanghai spent on average 1.69 hours on MVPA, 1.10 hours viewing screen, 1.27 hours doing homework, and 9.60 hours sleeping per day. Children who spent more time in screen viewing and less time in sleep have higher odds to be obese and those who spent less time in MVPA, sleep and more time in in screen viewing are more likely to have suboptimal mental health. Among all the activities, the magnitudes of sleep associated with both outcomes are the largest.

Using CLASS, a study in Australia reported that children aged 9–12 spent on average 2.14 hours daily on MVPA^37^. Another study in Hong Kong reported that children aged 9–14 spent 1.24 hours daily on MVPA^38^. As for screen viewing, Shanghai students spent more time than US students aged 6–11 (1.10 vs. 0.89 hours)^39^.

However, Shanghai students spent less time on sleep. A study comparing the sleep durations of primary school students showed that the sleep duration of Chinese children was 1 hour shorter than that of US children (9.25 vs. 10.15 hours)^40^. They also had a shorter sleep duration than British students (9.82 hours)^41^, but a longer sleep duration than their Hong Kong counterparts (8.79 hours)^42^. Overall, inadequate sleep has been a major concern among primary school students in Shanghai.

Extensive literature has linked each of the health behaviors we studied and health outcomes^43–48^. However, considering the 24-hour time limit, children face trade-offs when allocating their time. Longer screen time often implies shorter sleep duration or reduced homework time^24,49^, while more time doing homework may be linked to a later bedtime and shorter sleep duration^50^. The competing nature of these behaviors hinders our understanding of whether the association was resulted from the behavior itself or the displacement of another behavior. It is often difficult to disentangle the potential mechanisms behind the associations, which could be meaningful for developing effective interventions. To address this issue, several studies have investigated screen exposure and physical activity simultaneously. Studies have found that, compared with MVPA, screen time has a stronger association with BMI^51,52^. But long screen time and insufficient MVPA both independently predicted depressive and anxiety symptoms and school life dissatisfaction^53^. Sleep duration was also linked to obesity as strongly as sitting time and even more strongly than physical activity^54^. In our study, inadequate sleep was associated with the greatest odds for both obesity and poor mental health, which may reflect the problem of insufficient sleep among students in Shanghai. In fact, a local policy has been implemented in 2005 to delay school start times. This policy has increased students’ sleep duration from 9.35 hours per day in 2005^55^. However, the problem has not yet been fully addressed. More efforts from parents, schools, and communities are needed.

To better understand the associations between different time-use patterns and obesity and mental health, we performed an optimization analysis with total time constraints of the four activities ranging from 10 to 15 hours. It is noteworthy that, even though the current guidelines suggest that screen time should not exceed 2 hours per day, the increased time allocated to screen viewing would decrease the time spent in other beneficial activities. Based on the optimization results, screen time should be minimized to ensure enough sleep and allow for more time in healthy activities such as MVPA. This is also consistent with the latest AAP recommendations for children’s media use, which suggested that for children aged 6 and older, parents should place consistent limits on the time spent on screens, and make sure that screen time did not take the place of sleep, physical activity, or other behaviors beneficial for health^56^.

To our knowledge, this was the first study to report the combined association between MVPA, screen exposure, sleep, homework and BMI and mental health in primary school children. Many countries, including the US and UK, have shown increasing interest in understanding whether the top-ranked Shanghai education model also benefits students’ health^23^. The current study showed that certain time use patterns in Shanghai’s students were not optimal for health.

There were several limitations in this study. First, for sleep and MVPA, it would be more accurate to use objective measurement such as accelerometers. Self-reported information using questionnaires can suffer from social desirability bias, such as overestimating MVPA. And for screen exposure, CLASS-C did not include time that children spent with tablets and smartphones. The total screen time may be underestimated. Second, we chose BMI and mental health as the study outcomes and did not examine other issues that may also be of interest to the development of school-aged children, such as scholastic performance. Future studies are needed to evaluate the overall development of children. Third, the cross-sectional nature of the study restricted our potential to identify a causal link between daily time use and children’s health and did not rule out the possibility of reverse causality between the health behaviors and outcomes under study. Intervention studies would be warranted to clarify the causal pathways and illustrate the mechanisms between time use patterns and obesity and mental health.

The findings from this study suggested that students’ daily time allocation was related to their physical and mental health. Insufficient sleep and too much screen time were two major concerns among primary school students in Shanghai. Guidelines for school-aged children should consider the overall time use pattern to promote children’s health.

## Electronic supplementary material

Appendix
