# Supplementary material for: Daily Time-Use Patterns and Obesity and Mental Health among Primary School Students in Shanghai: A Population-Based Cross-Sectional Study
Source: Sci Rep. 2017 Nov 23;7:16200. doi: 10.1038/s41598-017-15102-4 (PMC5700943; doi:10.1038/s41598-017-15102-4)
Supplement: Supplementary file 1 — Appendix [file 41598_2017_15102_MOESM1_ESM.pdf]

Supplementary Document

Daily Time-Use Patterns and Obesity and Mental Health among Primary School Students in Shanghai: A Population-Based Cross-Sectional Study

Yunting Zhang, PhD, Donglan Zhang, PhD, Xinyue Li, MS, Patrick Ip, MD, Frederick Ho, MD, Yanrui Jiang, MD, Wanqi Sun, MD, Qi Zhu, MS, Weiming Zhu, MD, PhD, Jun Zhang, MD, PhD, Hongyu Zhao, PhD, Guanghai Wang, PhD, Xiaoming Shen, MD, PhD, Fan Jiang, MD, PhD

Appendix 1

Sampling procedures, Missing Value Patterns and Multiple Imputation

1. Sampling procedures

The Shanghai Children's Health, Education and Lifestyle Evaluation (SCHEDULE) was designed to investigate lifestyles including physical activity, sleep, nutrion and etc of Shanghai primary school students. This study also evaluated several health and educational outcomes including obesity, mental health, precocious puberty and academic performance. Since the incidence rate of precocious puberty was lowest among all health outcomes, a sample size of 16546 was calculated according to it. In 2014, we first obtained a complete list of primary schools in Shanghai with information on the number of students in each school from the Shanghai Education Commission, and selected the study participants using a multistage cluster sampling approach. Briefly, the sampling procedure started with the 16 districts and 1 county in the Shanghai Municipality. Chongming County and Pudong New District were purposely selected because they are significantly different from the other regions and have unique modernized urban planning. Five districts (3 urban, 1 peri-urban, 1 suburban) were then randomly selected from the remaining 15 districts in Shanghai (9 urban, 3 peri-urban, 3 suburban) using stratified

random sampling. In total, 6 districts and 1 county were selected to represent the whole city. Second, we randomly selected 26 public primary schools, the primary sampling units (PSUs), from the 7 districts/county in proportion to population size. Third, we stratified the sampled schools by school size. All students from grades 1 to 5 were selected if a school had less than 1000 students, and half of the students in a school were selected if the number of students was above 1000, with random selection of class numbers. The number of districts, schools are chosen according to the total sample size and stucture of each sampling stratas. Finally, we interviewed the sampled students aged 6–11 years and also interviewed one of their parents and the head teacher of their classes.

The onsite investigation was carried out by medical students in their 3rd or 4th year of study. We conducted training for the investigators and school administrators 2 weeks before the investigation. Onsite evaluation and questionnaire administration was completed within 2 weeks. A total of 54 persons participated in the data entry. To control for the quality of data entry, we randomly selected 10 questionnaires for double entry from each data entry person at the beginning of this process. The rate of entry error was within 0.06%–9.55%. For the five data entry persons whose error rate was over 5%, all of the data were double-entered and cross-checked.

2. Missing Value Patterns and Multiple Imputation

As shown in Figure S1, among the 17318 observations, 2.15% - 9.50% (n = 373 - 1645) had missing values on the items of the CLASS-C questionnaire, 2.38% - 14.28% (n=412 – 2473) contained missing values on the weight and height measures, 9.28% (n = 1608) had missing values on the SDQ measures, 4.47% (n=774) were missing on the sleep

duration measure, 7.98% (n=1382) were missing on the dietary patterns scores, and another 1.06% - 31.31% (n=184 - 5424) contained missing values in the covariates such as age, gender and socioeconomic status. Further cross tabulation analyses were performed to identify patterns of missing data. These analyses revealed that the missing data for most variables was at random, which showed that multiple imputations may be appropriate to handle the incomplete information.

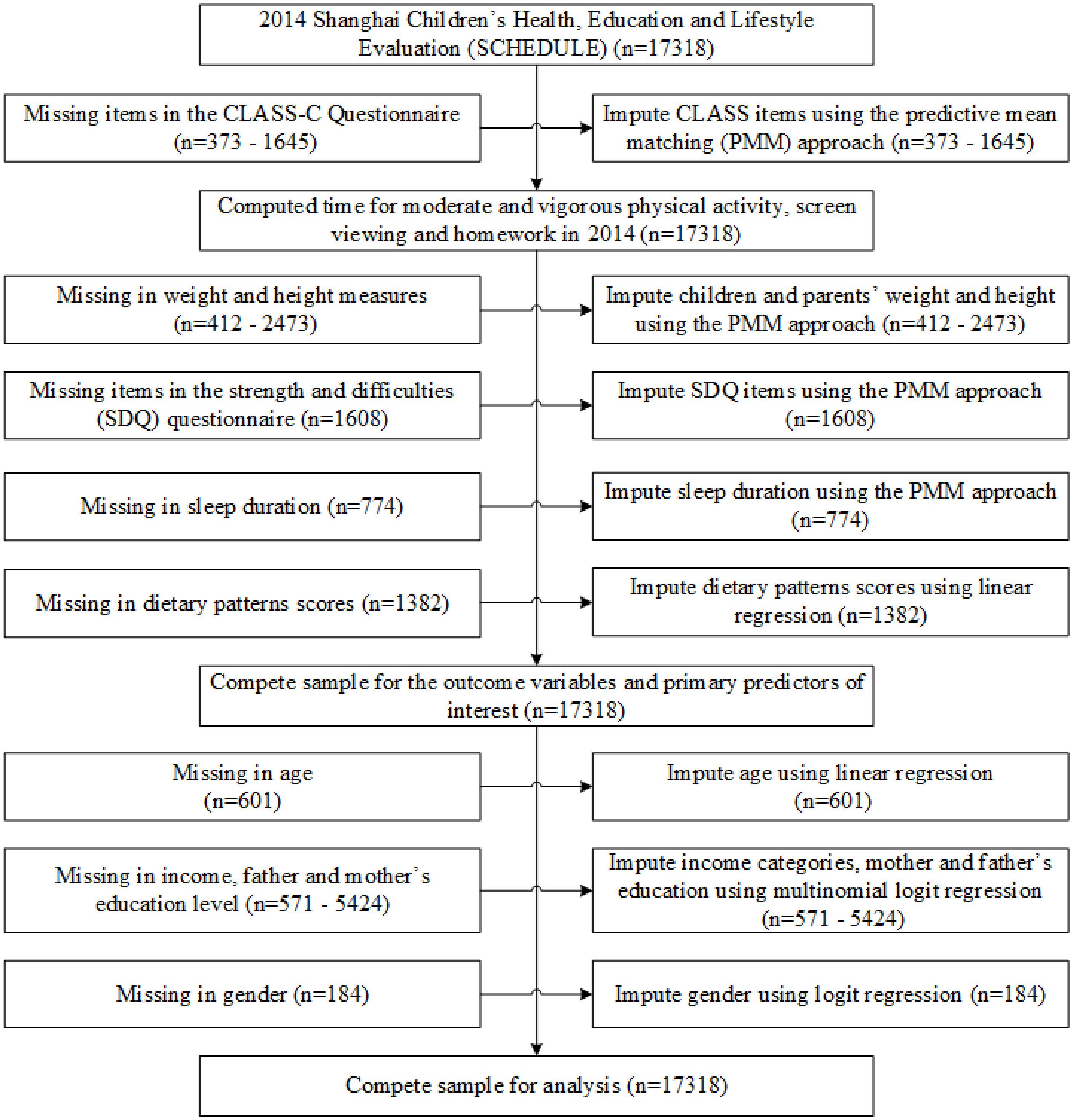

Figure S1. Missing Values in the Variables and Process of the Multiple Imputations to Construct the Final Analytic Sample (n=17318)

Table S1 Comparison of the observed characteristics between participants with complete and incomplete data (n= 17318)

Hours/day	Complete sample n= 8,806	Incomplete sample n=8,512	P-value [c]
**Time spent in each activity (mean (standard deviation))**			
Moderate to vigorous physical activity	1.54 (1.16)	1.41 (1.14)	<0.001
Screen time	1.06 (1.18)	0.99 (1.16)	<0.001
Sleep time	9.26 (0.63)	9.28 (0.68)	0.054
Homework time	1.36 (1.23)	1.17 (1.26)	<0.001
**Weight Status %** [a]			
Normal	89.03	89.33	0.568
Obese	10.97	10.67	
**Mental health %** [b]			
Normal	89.93	87.85	<0.001
Abnormal	10.07	12.15	
**Age group %**			<0.001
< 8	24.19	30.29	
8 – 10	40.82	40.93	
>= 10	34.99	28.78	
**Gender %**			<0.001
Male	52.42	55.16	
Female	47.58	44.84	
**Family monthly income %**			<0.001
< 30,000	8.70	10.36	
30,000 – 10,0000	32.51	31.88	
10,000 – 30,0000	27.19	23.33	
> = 30,0000	5.81	4.60	
Don’t know	25.79	29.83	
**Mother’s educational attainment %**			<0.001
Middle school and below	31.75	36.66	
High school	24.56	29.08	
College and above	43.69	34.25	

**Father's educational attainment %**			<0.001
Middle school and below	27.48	31.06	
High school	28.55	32.03	
College and above	43.97	36.91	
**Having health problems %**			0.214
No	81.77	82.50	
Yes	18.23	17.50	

a. Weight Status was defined based on BMI-for-age percentiles in Chinese children.
b. Mental health was measured by the validated Chinese version Strength and Difficulties Questionnaire (SDQ). Normal was defined as SDQ score <= 16, and abnormal was defined as SDQ score >= 17.
c. T-test was used to compare the mean difference; and chi-square test was used to compare the difference in distributions of the categorical variables.

Table S2 Associations between children's daily time use and weight and mental health status: results from logistic regressions using the complete sample (n= 8,806)[a]

	Obesity [b]		Suboptimal Mental Health [c]	
	OR	(95% CI)	OR	(95% CI)
Moderate to vigorous physical activity (Reference: 1–2 hours/day)				
< 1 hours/day	1.01	(0.84, 1.23)	1.33**	(1.11, 1.60)
≥ 2 hours/day	1.15	(0.92, 1.44)	0.80	(0.63, 1.03)
Screen viewing (Reference < 1 hour/day)				
1- 2 hours/day	1.23*	(1.01, 1.50)	1.19*	(0.98, 1.45)
≥ 2 hours/day	1.25*	(1.00, 1.59)	1.36**	(1.09, 1.69)
Sleep duration (Reference ≥ 10 hour/day)				
< 9 hours/day	1.79***	(1.32, 2.41)	2.05***	(1.55, 2.70)
9-10 hours/day	1.42***	(1.17, 1.72)	1.26*	(1.05, 1.52)
Homework (Reference < 1 hour/day)				
1- 2 hours/day	1.11	(0.90, 1.36)	0.63***	(0.51, 0.79)
≥ 2 hours/day	1.04	(0.85, 1.27)	0.97	(0.80, 1.18)

a. Population weights were used. Results were presented as odds ratio (95% confidence interval). Models estimated the association between time spent in each activity and obesity and mental health outcomes, adjusting for age, sex, annual household income, parent' highest education level, and health problems. In the obesity model, we further adjusted for father and mother's body mass index and children's diet-related factor scores.

* P< 0.05 ** P<0.01 *** P<0.001

Appendix 2

Data analysis for food frequency questionnaire (FFQ)

We included vegetables, fruits, red meat, white meat, aquatic product, snacks, soft drinks, dairy products and fried food, altogether 9 categories of food in our modified FFQ. To derive food patterns by using factor analysis, the FACTOR procedure in STATA using principal components analysis and orthogonal rotation was used to drive 2 uncorrelated factors. Factor loading for the 2 food factors (food patterns) and the names assigned to each pattern are presented in Table S3

Table S3 Factor loadings for 2 food patterns among 15936 children in SCHEDULE study [a]

**Food group**	**Factor loading**
Factor 1: healthy food pattern	
vegetables	0.6311
fruits	0.6753
red meat	0.6348
white meat	0.5909
aquatic product	0.6377
dairy products	0.3102
Factor 2: junk food pattern	
white meat	0.3635
snacks	0.7080
soft drinks	0.7707
fried food	0.6542

a. Food groups with absolute values <0.30 are excluded for simplicity

Appendix 3

Data analysis for two-objective optimization

Finally, we fitted Generalized Additive Models (GAM) using the same covariates as in the logistic regression models and performed two-objective optimization to find the optimal time allocation that minimizes the probability of mental health abnormality and that of obesity simultaneously. [1] Specifically, GAM is an extension of the logistic regression model and we fit the GAM model as:

$$g(\mathrm{E}(Y)) = \beta_0 + \sum_i \alpha_i x_i + \sum_j f_j(x_j)$$

Where the response variable Y, after logit transformation via the link function $g(\cdot)$, depends on the covariates $x_i{}'$s linearly and depends on each covariate x_j via some smooth function f_j. Here we chose $x_i{}'$s as the adjustment covariates (age, gender, annual household income, parents' highest educational attainment, and the presence of health problems) and $x_j's$ as the time spent in each activity (sleep, MVPA, screen exposure, and homework). The fitted smooth function f_j can be linear but not necessarily linear, allowing for a more relaxed assumption on the relationship between the outcome and the time spent in each activity (x_j). Therefore, the flexibility in GAM can help us better capture the association and obtain the optimal time allocation. We used the R package mgcv to fit GAM. [2]

Based on GAM results, we further employed two-objective optimization approach to obtain the optimal time allocation by minimizing the sum of the probability of mental health abnormality and the probability of obesity: $\mathrm{P}(Y_{MH}) + \mathrm{P}(Y_{Ob})$. We gave equal

weight to the two probabilities as we viewed the two outcomes equally important. In addition, we would like to consider the trade-off between activities: the increased time spent for one activity necessarily decreases the time spent for other activities. To take that into account, we set the time constraint that the sum of the four time variables (sleep, MVPA, screen, and homework) is fixed and then performed two-objective optimization. It is under the rationale that given fixed total time, we want to find the optimal way to use time. Further, because the total time of the four variables ranges between 10 to 15 hours in 90% of our data, we performed optimization under the sum constraint at 10, 11, ... , 15 hours respectively. We did not allow for zero values in the optimization procedure as recommending zero time for any activity is not realistic.

1. Hastie TJ, Tibshirani RJ. Generalized additive models. CRC Press, 1990.

2. Wood, SN. Fast stable restricted maximum likelihood and marginal likelihood estimation of semiparametric generalized linear models. Journal of the Royal Statistical Society 2011;73: 3-36.

Result from GAM analysis

Table S4. The GAM outputs for the two outcomes showing the statistical significance of the non-linear fits for the four time variables.

**Mental Health**		
	Chi.sq	p-value
s(mvpa)	26.673	<0.001
s(screen)	9.589	0.025
s(sleep)	55.623	<0.001
s(homework)	46.696	<0.001

**Obesity**		
	Chi.sq	p-value
s(mvpa)	0.077	0.782
s(screen)	18.262	<0.001
s(sleep)	31.234	<0.001
s(homework)	0.195	0.659
